# *Stat5b* Regulates Sexually Dimorphic Gene Expression in Zebrafish Liver

**DOI:** 10.3389/fphys.2018.00676

**Published:** 2018-05-31

**Authors:** Peipei Huang, Shuting Xiong, Jingliang Kang, Jie Mei, Jian-Fang Gui

**Affiliations:** ^1^State Key Laboratory of Freshwater Ecology and Biotechnology, Institute of Hydrobiology, Chinese Academy of Sciences, University of the Chinese Academy of Sciences, Wuhan, China; ^2^Key Laboratory of Freshwater Animal Breeding, Ministry of Agriculture, College of Fisheries, Huazhong Agricultural University, Wuhan, China

**Keywords:** sexual size dimorphism, stat5b, comparative transcriptome, gene expression, pathways

## Abstract

Sexual size dimorphism is an interesting phenomenon occurred in many fish species. Wildtype zebrafish exhibits a significant sexual dimorphism in body size at the adult stage. Previous studies indicated that sexual size dimorphism was eliminated in *stat5b*-mutated zebrafish. Herein, the comparative transcriptome analysis was conducted to observe the genes and pathways involved in sexual size dimorphism. The number of male-biased and female-biased genes was much less in the liver of *stat5b* mutant zebrafish than in wildtype. Gene ontology (GO) enrichment and Kyoto encyclopedia of genes and genomes (KEGG) pathway analysis indicated that multiple pathways related to metabolism were affected upon loss of *stat5b* function. qRT-PCR results also validated that sexually dimorphic expression of a set of genes was lost when *stat5b* was mutated. Furthermore, the weighted correlation network analysis (WGCNA) detected many candidate genes related to the growth traits and *stat5b* function, such as *greb1*, *lepr,* and *igf2b*. Our data suggest that *stat5b* should regulate the sexually dimorphic gene expression in zebrafish liver and add in understanding of the molecular mechanisms underlying sexual size dimorphism in fish species.

## Introduction

Sexual size dimorphism, the relative difference in body size and growth rate between male and female of the same species, has been reported in many fish species such as turbot (*Scophthalmus maximus*), Chinese tongue sole (*Cynoglossus semilaevis*), tilapia (*Oreochromis niloticus*) and yellow catfish (*Pelteobagrus fulvidraco*) (Gui and Zhu, 2012; Mei and Gui, 2015). In teleost fish, the body growth is usually controlled by the GH/IGF axis gene expressed in the hypothalamus-pituitary-gonad (HPG) axis (Rosenfeld and Hwa, 2009; Li and Lin, 2010; Rotwein, 2012), and the consequent Jak2/Stat5b signaling pathway activated by GH/GHR signal (Ahmed and Farquharson, 2010; Rotwein, 2012). Stat5b-deficient mammals were further revealed to have impaired body growth (Udy et al., 1997; Kofoed et al., 2003; Zhang et al., 2012).

STAT5b is an important transcription factor that plays critical roles in many biological processes such as cell proliferation, differentiation, reproduction, drug and lipid metabolism, and immune regulation (Grimley et al., 1999). *Stat5b* is mainly expressed in the liver and responds to the growth factor signals. GH has been shown to stimulate phosphorylation of JAK2 and activation of Stat5b transcriptional activity (Ahmed and Farquharson, 2010). GH-induced liver IGF-I gene expression was reduced upon loss of Stat5b function (Davey et al., 2001; Lau-Corona et al., 2017). Moreover, Stat5b has been reported to be involved in the regulation of body growth and adiposity in a sexual dimorphic pattern (Waxman and O’Connor, 2006; Oshida et al., 2016b; Nault et al., 2017). Growth analysis indicated that male mice grow faster than female mice. *Stat5b* is required for sexual size dimorphism and male-biased liver geneexpression in mouse, as *Stat5b* gene disruption leads to a significant reduction in the sexual size dimorphic phenotype and the male-biased gene expression in the liver (Udy et al., 1997; Zhang et al., 2012). Several master transcription factors including Stat5b and its downstream factors, B-cell lymphoma 6 (BCL6), cut-like homeobox 2 (CUX2) and Hepatocyte-enriched nuclear factor 6(HNF6) regulate the sex-biased gene expression in the liver of mice (Conforto et al., 2012, 2015; Zhang et al., 2012; Sugathan and Waxman, 2013). Although, male-biased and female-biased size dimorphism has been observed in many fish species, the functional studies of sexual size dimorphism remained scarce.

Previously, we found that stat5b (also known as *stat5.1*) regulates body growth in zebrafish. Moreover, female-biased size dimorphism was abolished in *stat5b*-mutated adult homozygous zebrafish (Xiong et al., 2017). In the present study, comparative transcriptome analysis was performed to assess the effect of *stat5b* deficiency on the sex-specific gene expression in zebrafish liver. Our findings reveal that *stat5b* disruption affects the expression of a subset of sex-dependent genes in the liver of male zebrafish, compared with that in female zebrafish. Our data suggest that Stat5b is an important transcriptional factor that regulates sexually dimorphic gene expression in zebrafish liver.

## Materials and Methods

### Zebrafish Maintenance

Zebrafish (Danio rerio) were reared at 28.5°C according to a standard protocol (Westerfield, 2000). All experiments involved zebrafish were approved by the institution animal care and use committee of Huazhong Agricultural University. *Stat5b*-deficient zebrafish was generated by CRISPR/CAS9 technology by our previous method (Xiong et al., 2017).

### Samples Collection and RNA Isolation

Liver samples from 3-month-old WT and *stat5b*-deficient zebrafish were harvested, snap frozen in liquid nitrogen and stored at -80°C until further use. Total RNA from the liver sample of zebrafish (livers of three individuals were combined into one sample) was isolated from each sample using SV Total RNA Isolation System (Promega, United States) with Dnase treatment according to the manufacturer’s protocol. Twelve samples were used in this study: three WT male liver samples (WM), three WT female liver samples (WFM), three *stat5b*-mutated male liver samples (SM) and three *stat5b*-mutated female liver samples (SFM). All the RNA samples were quantified and qualified by Agilent 2100 Bioanalyzer (Agilent Technologies, Palo Alto, CA, United States), NanoDrop (Thermo Fisher Scientific Inc.) and 1% agarose gel. 1 μg total RNA with RIN value above 7 was used for the following library preparation. Next generation sequencing library preparations were constructed according to the manufacturer’s protocol (NEBNext^®^ Ultra^TM^ RNA Library Prep Kit for Illumina^®^).

### RNA Sequencing Library Construction

PolyA+ mRNA was isolated from 1 μg total RNA using NEBNext^®^ Ultra RNA Library Magnetic Isolation Module. The libraries were constructed using the NEBNext Ultra RNA Library Prep Kit for Illumina, followed by purification with Agencourt AMPure XP beads (Beckman Genomics). Further, the concentration and quality of the library were assessed by Agilent bioanalyzer 2100. After automatically clustered using TruSeq PE Cluster Kit v4, the libraries were sequenced on Illumina HiSeq using paired-end protocol by GENEWIZ Biotechnology Co., LTD (Suzhou, China).

### Differential Expression Analysis

After filtering of the raw sequencing data with Trimmomatic-0.36 (Bolger et al., 2014), paired reads were mapped to the zebrafish genome (Zv10) using Hisat2 (Kim et al., 2015). Then the relative abundances of the transcripts were calculated using StringTie v1.3.1c (Pertea et al., 2015). RPM (reads per million reads mapped) generated by the -e parameter was extracted by a Python script (prepDE.py) in StringTie, then the RPM information would be processed by edgeR (version3.4.6) to estimate the differentiated expressed genes (Robinson et al., 2010). To characterize the genetic mechanism across body growth after the deletion of *stat5b*, we tested for pairwise differential expression among four group using edgeR, wildtype female (WFM) vs. wildtype male (WM), *stat5b*-mutated female (SFM) vs. *stat5b*-mutated male (SM), WFM vs. SFM and WM vs. SM. The transcripts would be filtered if they expressed at a minimum of CPM (one count per million) mapped reads (∼8–19 mapped reads per contig) in at least three of the 12 samples. The biological coefficient of variation (BCV) was estimated as the square root of the dispersion (McCarthy et al., 2012), and a multidimensional scale (MDS plot) was plotted using the 500 genes with higher dispersion among all samples. The heatmap was conducted using the expression level of the differentially expressed genes (DEGs) to figure out their expression pattern and clustering in all the samples. Genes with a false discovery rate (FDR) <0.05 based on Benjamini–Hochberg multiple test correction were identified as being differentially expressed. The following gene functions were annotated using gene ontology (GO) database and Kyoto encyclopedia of genes and genomes (KEGG) pathway using DAVID (version 6.8) (Huang et al., 2008; Sherman and Lempicki, 2009). The raw reads of these transcriptome data have been deposited to the NCBI database (accession no: SRR6327863-SRR6327874).

### WGCNA Analysis

To figure out the relationship between DEGs (from WFM vs. SFM and WM vs. SM) and growth traits (body length and body weight) (**Supplementary Table S1**), we introduced the R package WGCNA according to the previous description (Storey, 2002; Langfelder and Horvath, 2008). Expression correlation coefficients of all genes were calculated to select a suitable soft threshold to build gene networks using a scale-free topology model. Subsequently, gene expression modules with similar patterns were identified on the basis of gene cluster dendrogram and using the dynamic tree cut method (minModuleSize = 50 and mergeCutHeight = 0.25). To identify modules that were significantly associated with the trait of samples, the module eigengenes were calculated and correlated with weight and length (**Supplementary Table S1**). Modules with high correlation value and *P* < 0.05 were considered as significantly trait-related modules. The correlated genes, with *P* < 0.05 of weight and length were considered as the putative genes related with body growth.

### Validation of RNA-Seq Data via qRT-PCR Assay

The FPKM (fragments per kilobase of exon per million fragments mapped) was calculated by the parameter–F of StringTie, which would be used for the validation of our DEGs of transcriptomes by qRT-PCR. Then 20 DEGs were randomly selected and subjected to qRT-PCR. Initially, the total RNAs were reversely transcripted into cDNA by GoScript^TM^ Reverse Transcription System (Promega) according to the manual protocol. All the qRT-PCR reactions were performed in a total volume of 20 μL using iTaq^TM^ Universal SYBR Green Supermix (Bio-Rad, United States), as previously described (Jing et al., 2015). Specificity of amplification for each reaction was determined by dissociation curves and gel electrophoresis. Each experiment was performed in triplicate and the data were analyzed using the 2^-ΔΔCt^ formula. The relative expression of target genes was normalized to the expression of *ef1a*. Primers used in this process are shown in **Supplementary Table S2**.

## Results

### Analysis of the Transcriptome Data and Relative Similarity of Liver Samples Between the Wildtype and stat5b-Mutated Zebrafish

To characterize the differential gene expressions in the *stat5b*-mutated liver of zebrafish, we performed transcriptome analysis using a HiSeq sequencing platform. Three biological replicates were carried out for each experiment. All raw reads and quality control statistics of the sequencing data are presented in **Supplementary Table S3**. The sequencing data of 12 liver libraries generated a mean of 61,038,747 raw reads, of which a mean of 60,108,888 reads (98.48%) with Q20 value above 95% after quality filtering. All the high-quality reads were subsequently mapped to the zebrafish genome and assembled into transcripts. In total, we identified 37,019 assembled transcripts that were used for following DEG analysis.

**FIGURE 1:**
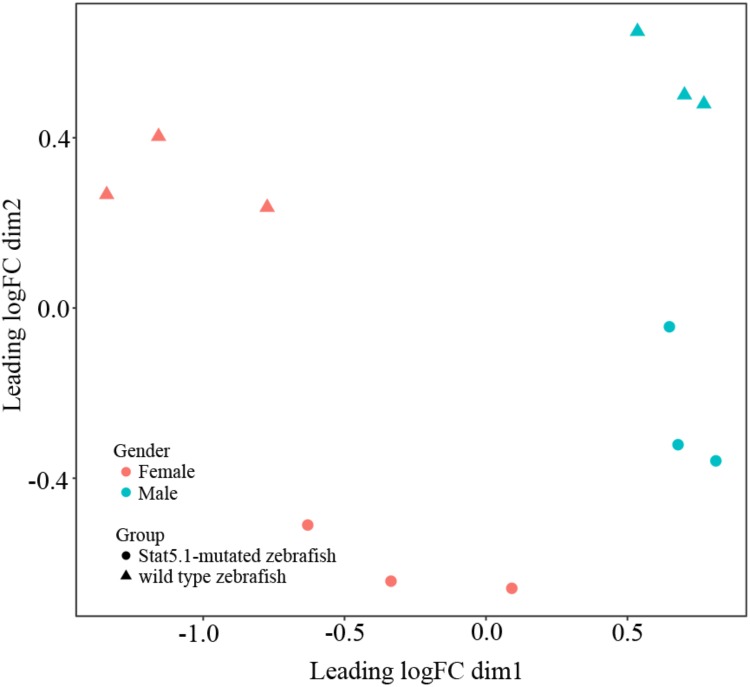
Multidimensional scaling analysis (MDS) representing the distances between wildtype and *stat5b* mutant samples. Dimensions (Dim) 1 and 2 represent distances between samples, which was calculated by average log2 fold change for the top 500 genes. Dim 1 showed the difference between male and female and dim 2 showed the difference between stat5b-mutated and wildtype samples. The triangle represented wildtype samples and the circle represented *stat5b*-mutated samples.

To visualize the relationships within the transcriptomes, we carried out a correlation analysis using a multidimensional scaling (MDS) plot. As shown in **Figure 1**, all the twelve samples were clustered into four groups that were consistent with our assigned groups, confirming the veracity of the biological repeats in each group. The distance between wildtype male and female on the MDS plot (X axis) reflected the sexually dimorphic gene expression in the liver of zebrafish. Interestingly, the distance between *stat5b*-mutated male and female was much closer than that of wildtype male and female, suggesting that the sexually dimorphic gene expression in zebrafish liver was reduced when the function of *stat5b* was lost.

### Global Changes in Gene Expression in Zebrafish Liver Upon Loss of stat5b Function

**FIGURE 2:**
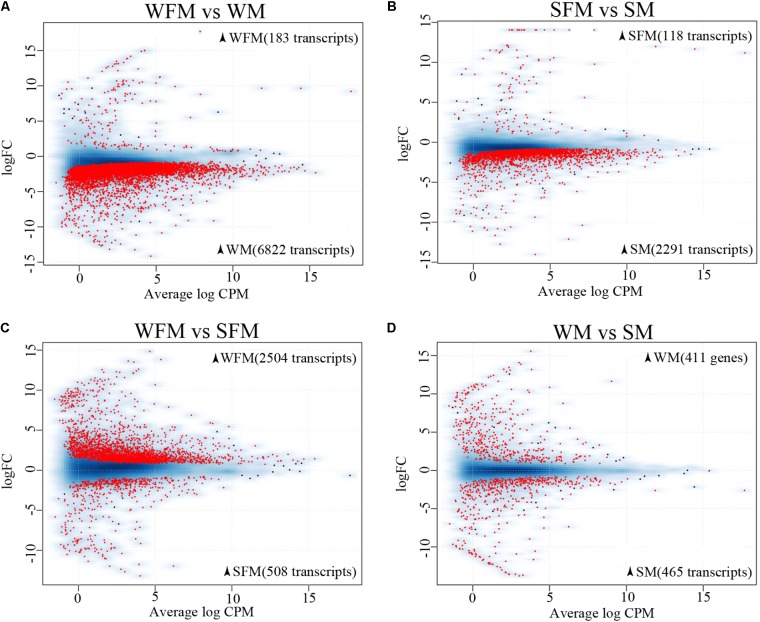
Global analysis of gene expression profiling. **(A–D)** Two-dimensional scatter diagram showing the number of differentially expressed genes (FDR <0.5) in red in each pairwise comparison. Wildtype female (WFM), wildtype male (WM), *stat5b*-mutated female (SFM), *stat5b*-mutated male (SM). A smooth scatter was used to convert the number of points in each plot coordinate into a vector of colors representing the local point density. Darker shades of blue represent higher density of point. Only below a specific density or in case of significance the dot is drawn. The numbers of sex-biased genes in the respective type of fish were shown in brackets.

There were 7,005 differentially expressed transcripts between wildtype males and females, of which 6,822 transcripts were male-biased and 183 transcripts were female-biased (**Figure 2A**). However, there were 2,409 differentially expressed transcripts between *stat5b*-mutated males and females, of which 2,291 transcripts were male-biased and 118 transcripts were female-biased (**Figure 2B**). These data suggest that *stat5b* regulate the sexually dimorphic gene expression in the liver of zebrafish, as the male-biased and female-biased transcripts were greatly reduced in *stat5b*-mutated zebrafish compared to wildtype. Comparing gene expression between wildtype and stat5b-mutated females, there were 3,012 differentially expressed transcripts, of which 2,504 and 508 transcripts were highly expressed in wildtype and *stat5b* mutants, respectively (**Figure 2C**). Between wildtype and stat5b-mutated males, 411 and 465 differentially expressed transcripts were highly expressed in wildtype and *stat5b* mutants, respectively (**Figure 2D**). The detailed information is shown in **Supplementary Table S4**.

**FIGURE 3:**
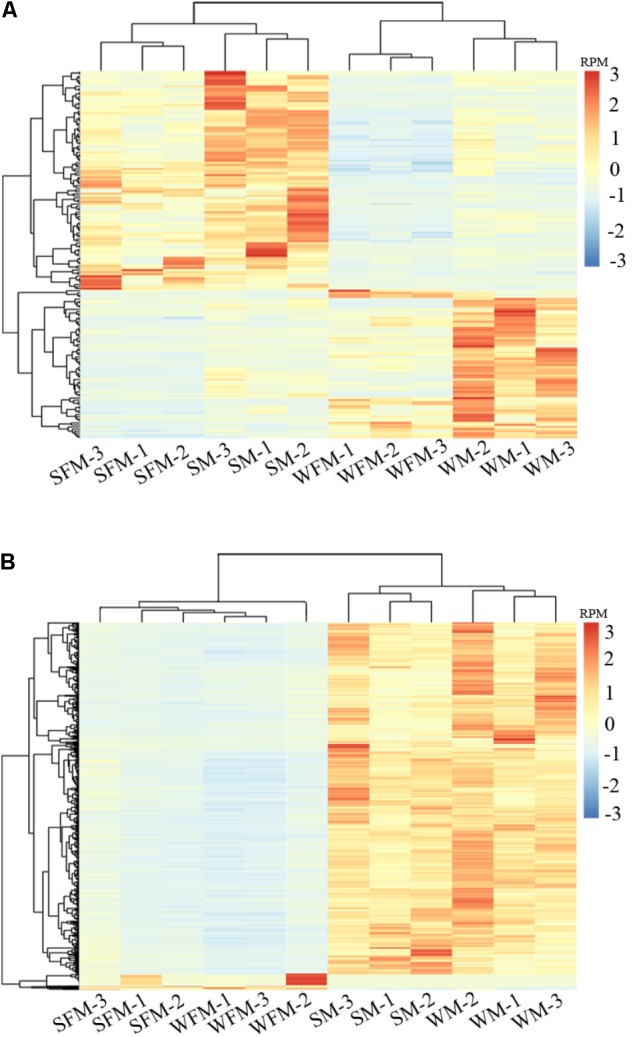
Differential gene expression analysis and clustering. Heat map of the DEGs among four comparisons (WFM vs. WM, SFM vs. SM, WFM vs. SFM, and WM vs. SM). **(A)** Common DEGs of WFM vs. SFM and WM vs. SM; **(B)** Common DEGs of WFM vs. WM and SFM vs. SM. Colors represent the RPM (reads per million reads mapped) of the genes expression after scaling and centering.

To further validate the reliability of the DEGs, heatmaps were constructed to visualize expression patterns of DEGs in wildtype and *stat5b*-mutated female and male zebrafish (**Figure 3**). 183 common genes were observed in the DEGs of WFM vs. SFM and WM vs. SM (**Supplementary Table S5**), which indicated that knockout of *stat5b* would affect the expressions of these genes no matter the sex. The heatmap results showed that all samples were separated as two groups, wild type (WFM and WM) and mutants (SFM and SM), which indicated the expression of these DEGs was apparent different after loss of *stat5b* function. In addition, all the three biological repeats of the same samples were clustered into one branch, suggesting the reliability of the biological repeats in each group (**Figure 3A**). To identify the gene expression differentiation owing to sex, we combined the DEGs of WFM vs. WM and SFM vs. SM and found 1,569 common genes in these two datasets. The heatmap exhibited two distinct main clusters (Male and Female) and most genes had a male-biased expression pattern (**Figure 3B**). We also generated a simplified heatmap that summed the RPM value of three replicate by R package (**Supplementary Figure S1**), which showed a similar expression pattern as **Figure 3**.

### Pathways Enrichment Analysis of Differentially Expressed Genes (DEGs) Between Wildtype and *stat5b* Mutant Zebrafish Livers

**FIGURE 4:**
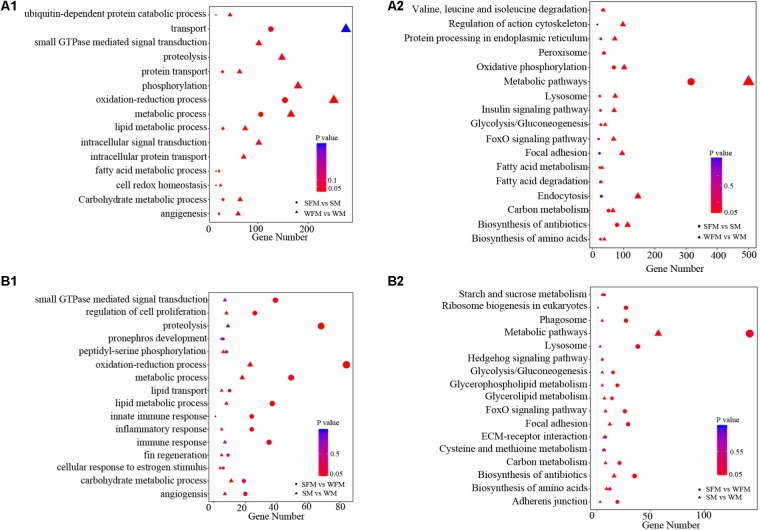
The GO terms enrichment **(A1,B1)** and KEGG pathway **(A2,B2)** analyses of DEGs among different comparisons. The X-axis (gene number) indicates the number of DEGs in each GO functions and KEGG pathway. The color and size of dot represent *P*-value and number of DEGs assigned to the corresponding GO term and KEGG pathway, respectively.

**FIGURE 5:**
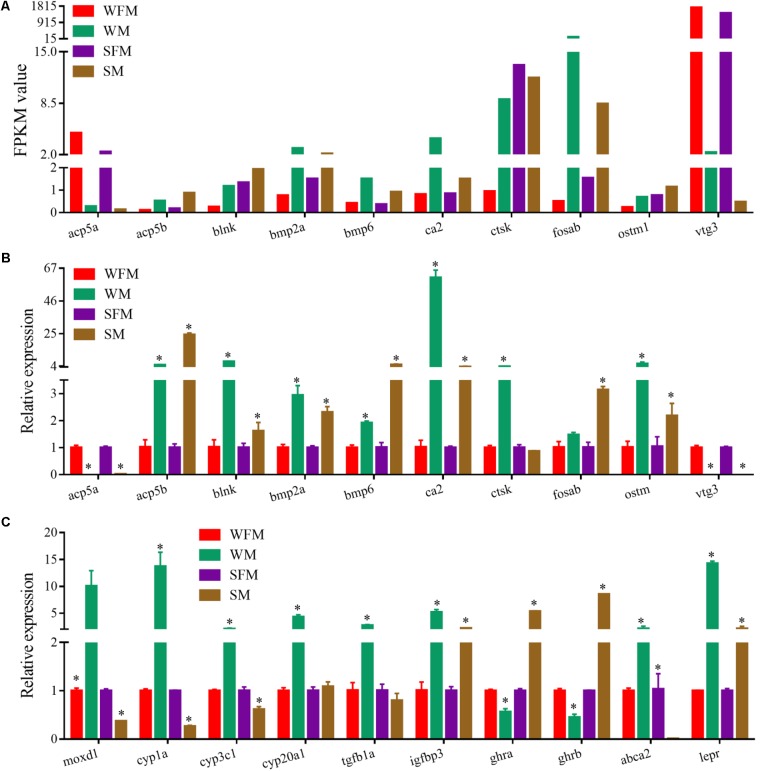
Verification of the transcriptome data by qRT-PCR. **(A)** Profile of Illumina sequencing value for selected genes with normalized expression data (FPKM value). **(B,C)** Relative expression of selected genes by qRT-PCR. Values are indicated as means ± standard deviation (SD). The data was assessed using a Student’s *t*-test. A probability *P* < 0.05 was considered statistically significant. ^∗^*P* < 0.05.

To further explore the molecular functions of DEGs between wildtype and *stat5b* mutant liver of zebrafish, we introduced a comparative GO analysis (**Figure 4**) and KEGG analysis (**Figure 5**). In the results of GO analysis, the DEGs in both WFM vs. WM and SFM vs. SM were enriched in protein transport, oxidation-reduction process, metabolic process, lipid metabolic process and carbohydrate metabolic process. Meanwhile, we observed that the number of DEGs in several GO processes dramatically reduced in *stat5b*-mutated zebrafish, such as transport, small GTPs mediated signal transduction, proteolysis, phosphorylation, oxidation-reduction process, metabolic process, lipid metabolic process, intracellular signal transduction, and intracellular protein transport. (**Figure 4A1**). After analyzing the difference between SFM vs. WFM and SM vs. WM, we found that most DEGs in SFM vs. WFM and SM vs. WM were enriched in metabolic process, regulation of cell proliferation and oxidation-reduction process (**Figure 4B1**).

Kyoto encyclopedia of genes and genomes analysis was performed to identify pathways, in which the DEGs were involved. The DEGs found in WFM vs. WM were most enriched in metabolic pathways and some other pathways including endocytosis, oxidative phosphorylation, biosynthesis of antibiotics, focal adhesion, lysosome, foxO signaling pathway, insulin signaling pathway, protein processing in endoplasmic reticulum and regulation of action cytoskeleton (**Figure 4A2**). In addition, the DEGs found in SFM vs. SM were most enriched in metabolic pathways, lysosome, ribosome biogenesis in eukaryotes, phagosome, foxO signaling pathway, glycolysis/gluconeogenesis, glycerophospholipid metabolism, carbon metabolism, biosynthesis of antibiotics, and adherens junction (**Figure 4A2**). After comparing the involved KEGG pathways between WFM vs. WM and SFM vs. SM, we only found four common KEGG pathways in the top enrichment including metabolic pathway, biosynthesis of antibiotics, lysosome, and foxO signaling pathway. Moreover, the number of DEGs in these four KEGG pathways dramatically decreased upon the mutation of *stat5b*.

When analyzing the difference between SFM vs. WFM and SM vs. WM, we found that most DEGs in SFM vs. WFM were enriched in metabolic pathways, lysosome, ribosome biogenesis in eukaryotes, biosynthesis of antibiotics, phagosome, foxO signaling pathway, adherens junction, carbon metabolism, glycerophospholipid metabolism and glycolysis/gluconeogenesis. However, most DEGs in SM vs. WM were enriched in metabolic pathways, biosynthesis of antibiotics, focal adhesion, biosynthesis of amino acids, cysteine and methionine metabolism, carbon metabolism, ECM-receptor interaction, glycerolipid metabolism, hedgehog signaling pathway and starch and sucrose metabolism (**Figure 4B2**).

### Loss of Sexually Dimorphic Liver Gene Expression Upon Deletion of *stat*5b

qRT-PCR was performed to validate the transcriptome results on randomly selected genes, such as *acp5a*, *acp5b*, *blnk*, *bmp2a*, *bpm6*, *ca2*, *ctsk*, *fosab*, *ostm1,* and *vtg3*, which showed a sexually dimorphic gene expression profile in the transcriptome (**Figure 5A**). qRT-PCR results confirmed that the relative expression levels of these genes were consistent with the transcriptome data (**Figure 5B**). Meanwhile, we found that a number of genes changed their expression pattern when *stat5b* was mutated. As shown in **Figure 5C**, some male-biased genes such as *moxd1*, *cyp1a*, *cyp3c1*, *cyp20a1* changed their expression patterns in *stat5b* mutants. The expression levels of *moxd1*, *cyp1a*, *cyp3c1,* and *cyp20a1* in wildtype male were 10-, 13.7-, 2.1-, and 4.3-fold to that in wildtype female, whereas their expression in *stat5b* mutant male were 0.371, 0.222, 0.615, and 1.08 fold to that in mutant female, respectively. Moreover, expression levels of growth-related genes such as *tgfb1a*, *igfbp3*, *ghra*, *ghrb*, *abca2,* and *lepr* displayed a great change upon *stat5b* mutation. The male-biased expression of *tgfb1a*, *igfbp3,* and *lepr* in wildtype was significantly reduced in *stat5b* mutants. Interestingly, the expression of *abca2* was male-biased in wildtype but female-biased in the mutants. However, *ghra* and *ghrb* showed female-biased expression in wildtype and male-biased expression in the *stat5b* mutants (**Figure 5C**).

### WGCNA Analysis and Identification of Candidate Genes Associated With Growth Traits

Weighted correlation network analysis analysis was conducted to further investigate the relationship between sexual dimorphism and *stat5b* function. We use the DEGs in two groups (WFM vs. SFM and WM vs. SM) for following WGCNA analysis. Based on the correlation coefficients of DEGs between wildtype and *stat5b*-mutated liver, cluster dendrogram was constructed in female and male with a power value = 14 (**Supplementary Figures S2A,B**).

**FIGURE 6:**
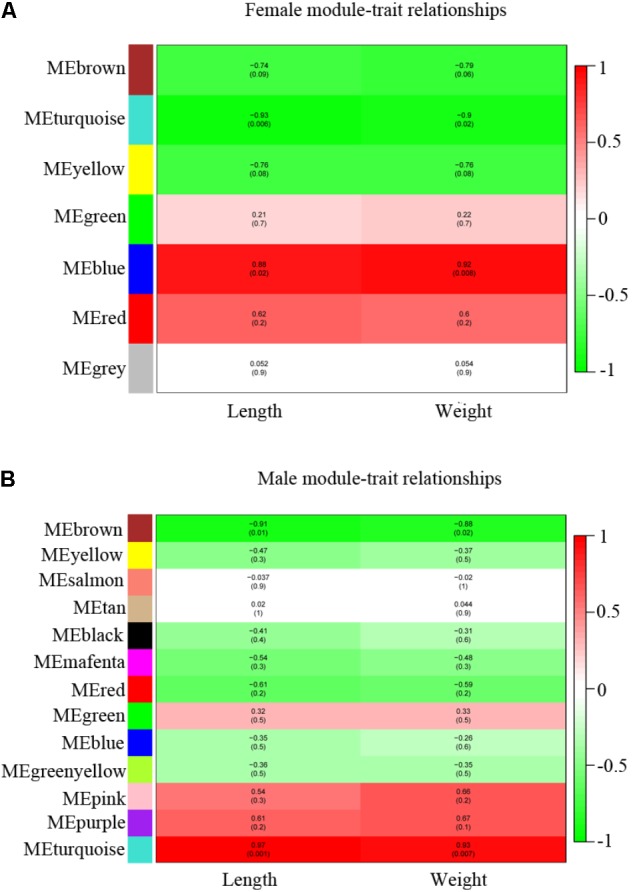
WGCNA identification of gene modules correlated with growth traits (length and weight). **(A,B)** Heatmap of modules associated with growth trait in female **(A)** and male **(B)**. Each row corresponds to a module eigengene and column to a trait. Each cell contains the corresponding correlation and *p*-value. The table is color-coded by correlation according to the color legend, red (positive correlation) and green (negative correlation).

We calculated the correlation coefficient between module membership and potential genes for growth traits (length and weight) (**Supplementary Table S1**) to identify the trait-related modules. The gene modules were classified and clustered by similarity = 0.8 and minModuleSize = 50. As a result, 7 modules and 13 modules were identified in female and male, respectively (**Figures 6A,B** and **Supplementary Table S6**). The blue and turquoise modules were significantly positively correlated with growth traits in female and male, respectively (*p* < 0.05) (**Figures 6A,B** and **Supplementary Figures S3A–D**). There were 414 genes found in blue module screened in female and 512 genes were found in turquoise module in male individual (**Supplementary Table S6**). The red modules in female, pink and purple in male also exhibited a positive correlation with growth straits, although no significant difference (*p* > 0.05) was observed. We also filtered the data with poor positive correlation with growth straits and module (*p* > 0.05) in female blue module and male turquoise module and combined the difference between wildtype and stat5b mutants in each sex.

**FIGURE 7:**
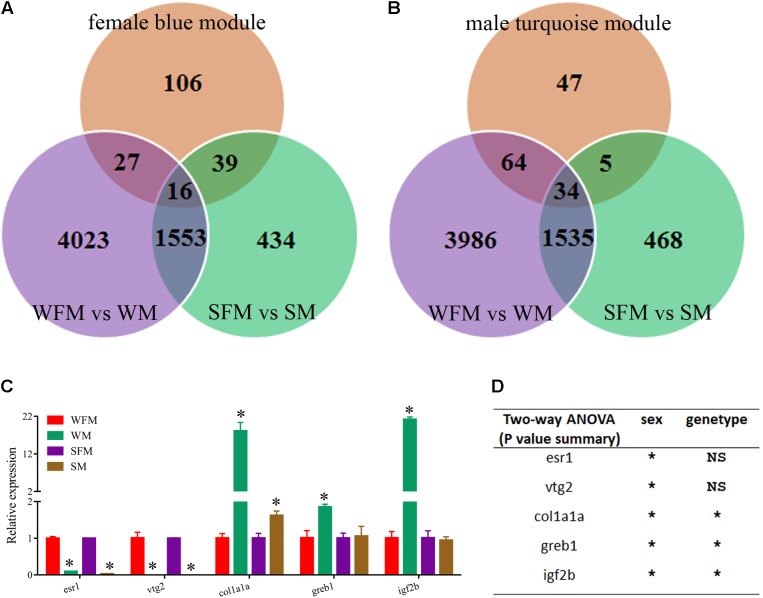
Identification of candidate genes correlated with growth traits. **(A)** Venn diagram of the candidate DEGs among three comparisons: WFM vs. WM (purple circle), SFM vs. SM (green circle) and growth traits-related candidate genes in female blue module (orange circle). **(B)** Venn diagram of the candidate DEGs among three comparisons: WFM vs. WM (purple circle), SFM vs. SM (green circle) and growth traits-related candidate genes in male turquoise module (orange circle). **(C)** qRT-PCR verification of several candidate genes related to growth traits, including *esr1*, *vtg2*, *clo1a1a*, *greb1,* and *igf2b*. Ef1a was used for the internal reference. **(D)** Two-way ANOVAs of mRNA expression levels of *esr1*, *vtg2*, *clo1a1a*, *greb1,* and *igf2b*. Values are indicated as means ± standard deviation (SD). All the experiments were conducted in triplicate. The data was assessed using a Student’s *t*-test and two-way ANOVA. Asterisk denoted significant differences and a probability *P* < 0.05 was considered statistically significant. NS, not significant. (^∗^*P* < 0.05; NS, *P* > 0.05).

Finally, we combined the sexual difference with these results and screened several candidate genes that were closely related with growth trait and sexual dimorphism. As shown in **Figures 7A,B**, the venn diagrams showed the candidate genes found in the growth-traits modules. We found five growth traits-related genes (*esr1*, *vtg2*, *col1a1a*, *greb1*, and *igf2b*) from these candidate genes and confirmed their expression pattern by qRT-PCR. As shown in **Figure 7C**, the relative expression levels of *esr1*, *vtg2*, *col1a1a*, *greb1*, and *igf2b* in wildtype male were 0.097-, 0.001-, 18.1-, 1.9-, and 21.2-fold to their expression in wildtype female, whereas their expression in *stat5b*-mutated male were 0.029-, 0.000002-, 1.6-, 1.1-, and 0.945-fold to that in *stat5b*-mutated female. The sex and stat5-mutated factor both significantly influence the expression levels of col1a1a, greb1, and igf2b (*P* < 0.05) (**Figure 7D**). Furthermore, these varied gene expression indicate that sexually dimorphic gene expression in zebrafish liver was reduced by loss of *stat5b* function.

**FIGURE 8:**
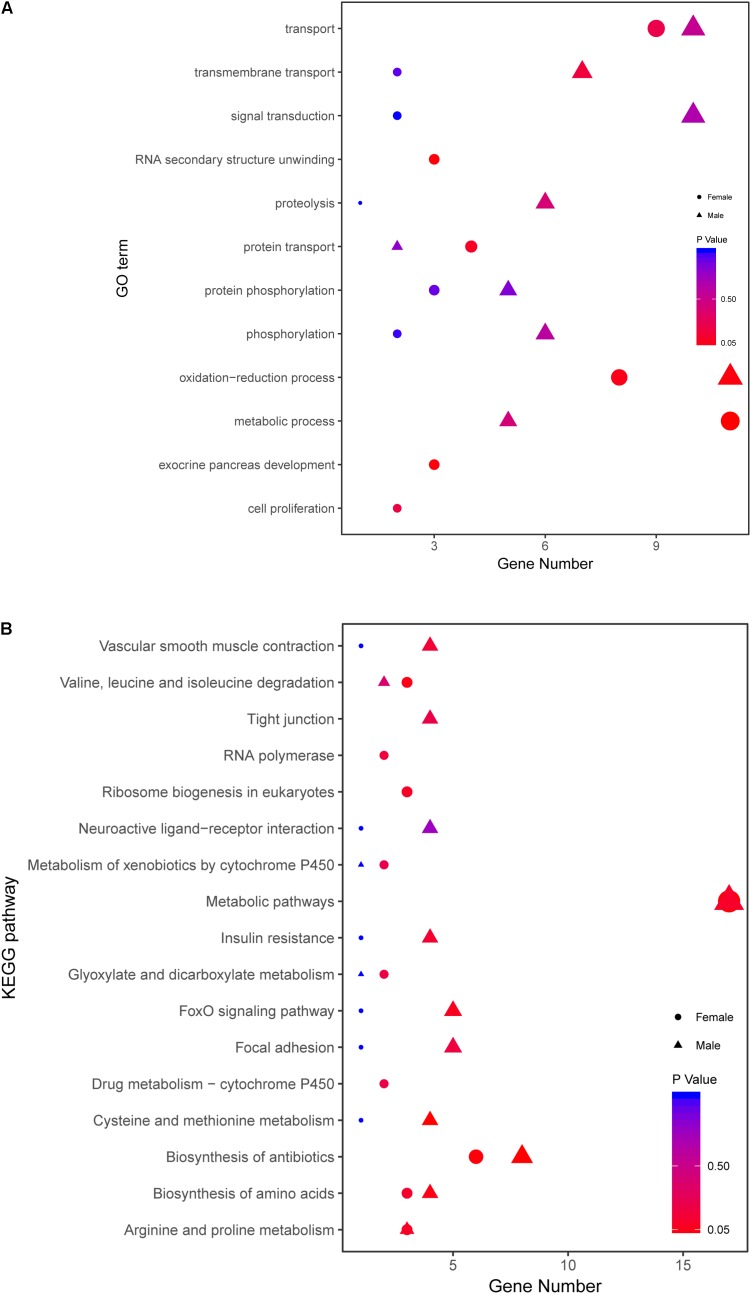
Comparative GO and KEGG analysis of growth traits-related genes between female and male. **(A)** GO enrichment analysis of growth traits-related DEGs in WFM vs. WM and SFM vs. SM. **(B)** KEGG analysis of growth traits-related DEGs in WFM vs. WM and SFM vs. SM. Wildtype female (WFM), wildtype male (WM), *stat5b*-mutated female (SFM), *stat5b*-mutated male (SM).

There are 82 genes screened in female and 103 genes in male zebrafish. The detailed information is listed in **Supplementary Table S7**. Then all the related genes in different sexes were annotated with GO and KEGG analysis (**Figure 8**). The female-related candidate genes were enriched in metabolic process, transport, oxidation-reduction process, protein transport, RNA secondary unwinding and exocrine pancreas development. However, the male-related ones were enriched in oxidation-reduction process, transport, signal transduction, transmembrane transport, proteolysis, phosphorylation, and metabolic process (**Figure 8A**). After KEGG analysis, both the female- and male-related genes were enriched in metabolic pathway, biosynthesis of antibiotics and biosynthesis of amino acids. The number of sex-related genes involved in metabolic pathway was similar between sexes (**Figure 8B**).

## Discussion

Sexual dimorphism in vertebrates mainly results from the evolution of sex-biased genes and their expression (Ellegren and Parsch, 2007; Williams and Carroll, 2009; Parsch and Ellegren, 2013). Sexual size dimorphism has been reported in many fish species, in which monosex fish production has been successfully established (Wang et al., 2009; Dan et al., 2013; Mei and Gui, 2015). However, the molecular mechanism of sexual size dimorphism is still unclear in fish species. In mouse, *Stat5b* is a critical regulator of male-biased size dimorphism (Udy et al., 1997). Previous studies indicated that *stat5b* (also known as *stat5.1*) control somatic growth and is involved in sexual size dimorphism in adult zebrafish (Xiong et al., 2017). To explore the molecular mechanism underlying sexual size dimorphism, we performed a comparative transcriptome analysis on wildtype and *stat5b*-mutated (both sexes) to characterize the function of *stat5b* and its downstream genes and pathways. High Q20 values, coherent MDS plot and clustering analyses confirmed high quality and good repeatability of the sequencing data (**Supplementary Table S3** and **Figure 2**).

Body growth in animals is usually regulated by expression of GH/IGF axis gene (Wang et al., 1999; Yakar et al., 1999; Lupu et al., 2001; Stratikopoulos et al., 2008). The sexually dimorphic gene expression in the liver is because of the differential influences of synthesized estrogens and androgens (Roy and Chatterjee, 1983). In Nile tilapia with male growth advantage, 17-Alpha-methyltestosterone (MT) treatment activated the GH/IGF axis genes and promoted somatic growth (Riley et al., 2002). Interestingly, in European eel [*Anguilla anguilla* (L.)] and half-smooth tongue sole with female growth advantage, and yellow catfish with male growth advantage, the expression of *GH*, *IGF-1* and *IGF-2* were significantly higher in the females and males that have faster growth rate, respectively (Degani et al., 2003; Ma et al., 2011, 2012, 2016). Zebrafish *stat5b* has been shown to transcriptionally regulate *gh1* gene expression. In addition, *ghra* was also detected in the CHIP-seq data of *stat5b* (Xiong et al., 2017). *Ghra* and *ghrb* have female-biased expression in wildtype zebrafish, in which female grows faster than male. In contrast, *ghra* and *ghrb* have male-biased expression in *stat5b*-mutated zebrafish that has no sexual size dimorphism (**Figure 5C**). *Igf2b* and greb1 have male-biased expression in wildtype zebrafish, whereas there were no expression difference between female and male in *stat5b*-mutated zebrafish (**Figure 7C**) (Li et al., 2017). Our data suggest that *stat5b* may control sexual size dimorphism in zebrafish by regulating the expression of GH/IGF axis gene.

*Ghrelin* and its receptor, growth hormone secretagogue receptor (*GHSR*) have been revealed to regulate feeding and GH/IGF signaling in vertebrates (Tschop et al., 2000; Nakazato et al., 2001). *Ghrelin* and *GHSR* showed male-biased expression in yellow catfish (Zhang et al., 2016). As an anorexigenic peptide hormone, *leptin* not only regulates the expression of GHR and IGFs to control somatic growth (Won et al., 2016), but also serves as a mediator of normal food intake and metabolism (Douros et al., 2017). In yellow catfish with male growth advantage, the expression of *leptin* and its receptor *lepr* were higher in female than in male (Zhang et al., 2017). In contrast, the expression of *lepr* in male wildtype zebrafish was higher than female wildtype zebrafish. The sexually dimorphic expression of *lepr* was reduced upon mutation of *stat5b*. These data suggest that *stat5b* may control sexual size dimorphism in zebrafish by regulating the expression of feeding-related gene.

Mouse Stat5b mutation led to a significant growth defects and affected the hepatic gene expression in a sexual dimorphism pattern (Udy et al., 1997; Oshida et al., 2016a). Interestingly, Stat5a and Stat5b have two distinct, non-overlapping functions in mediating GH regulation of sex-biased hepatic gene expression. Stat5a predominantly regulated female-biased hepatic genes in female liver while Stat5b primarily regulated male-biased genes in male liver though it also plays a role in female (Park et al., 1999; Clodfelter et al., 2006, 2007; Lamba et al., 2014). In addition, Stat5a and Stat5b are involved in the regulation of the genes involved in hepatic drug response and promote sexual dimorphism by regulating the expression of hepatic cytochromes P450 (CYPs) (Lamba et al., 2016). Hepatic CYPs could synthesize or metabolize endogenous and exogenous substances (Baldwin et al., 2009) and are involved in many pathways, such as drug metabolism and cholesterol biosynthesis (Rozman et al., 1996; Uno et al., 2012; McMillan and Tyndale, 2017). CYP genes have a sexual dimorphic expression pattern and might play a role in sexual size dimorphism (Zheng et al., 2013; Xiong et al., 2015). In this study, the male-biased expression of *cyp1a*, *cyp3c1,* and *cyp20a1* in wildtype zebrafish liver were disrupted when *stat5b* was mutated (**Figure 5C**). Although a pulsatile release of GH is existed in rodents, no similar phenotype was reported in fish species, suggesting the possible differences in molecular mechanisms of body growth between rodents and fish. In addition, after comparing the 183 common DEGs in WFM vs. SFM and WM vs. SM in zebrafish livers (**Supplementary Table S5**) and the common sex-biased DEGs between wildtype and Stat5b-null mouse livers (Oshida et al., 2016a,b), we did not found any overlapped common genes between zebrafish and mouse, which may hint that different molecular mechanisms were existed between male-biased and female-biased sexual size dimorphism. In summary, *stat5b* regulates sexually dimorphic gene expression in zebrafish liver, while the functions of its downstream genes in sexual size dimorphism in fish, need further investigation.

## Author Contributions

PH and SX performed the experiments, analyzed the data, drafted and revised the article critically, finally approved the version to be published, and agreed to be accountable for all aspects of the work in ensuring that questions related to the accuracy or integrity of any part of the work are appropriately investigated and resolved. JK participated in the work, analyzed the data, revised the report critically, and finally approved the version to be published. JM participated in designing the work, critical revision of the article, approval of the version to be published, and agreed to be accountable for all aspects of the work in ensuring that questions related to the accuracy or integrity of any part of the work are appropriately investigated and resolved. J-FG participated in designing the work, critical revision of the article, approval of the version to be published, and agreed to be accountable for all aspects of the work in ensuring that questions related to the accuracy or integrity of any part of the work are appropriately investigated and resolved.

## Conflict of Interest Statement

The authors declare that the research was conducted in the absence of any commercial or financial relationships that could be construed as a potential conflict of interest.

## Supplementary Material

FIGURE S1 Differential gene expression analysis and clustering. Heat map of the DEGs among four comparisons (WFM vs. WM, SFM vs. SM, WFM vs. SFM, and WM vs. SM). **(A)** Common DEGs of WFM vs. SFM and WM vs. SM; **(B)**. Common DEGs of WFM vs. WM and SFM vs. SM. Colors represent the summed RPM of three replicates after scaling and centering.

FIGURE S2 Cluster dendrogram with the WGCNA approach based on the correlation coefficients of DEGs between wildtype and *stat5b*-mutated livers in female **(A)** and male **(B)**. The height shows the distance between two separate genes. Branches in the cluster dendrogram indicate the divided modules based on gene clustering result. Colored modules indicate the membership of separate module by combining modules with similar expression patterns.

FIGURE S3 Module Membership vs. gene significance for growth trait. **(A)** Female module membership in blue module vs. gene significance for body length. **(B)** Female module membership in blue module vs. gene significance for body weight. **(C)** Male module membership in turquoise module vs. gene significance for body length. **(D)** Male module membership in turquoise module vs. gene significance for body weight.

TABLE S1 The growth traits (body length and body weight) of 3-month-old zebrafish for transcriptome.

TABLE S2 The information of primers used for qRT-PCR.

TABLE S3 Summary of the sequencing data. The number 1, 2, and 3 represent three replicate samples of each group.

TABLE S4 List of differentially expressed genes in WFM vs. WM, SFM vs. SM, WFM vs. SFM and WM vs. SM. Wildtype female (WFM), wildtype male (WM), *stat5b*-mutated female (SFM) and *stat5b*-mutated male (SM).

TABLE S5 Detailed information of common DEGs in WFM vs. SFM and WM vs. SM.

TABLE S6 Detailed information of candidate genes related to growth traits in both sexes.

TABLE S7 Detailed information of candidate genes related to growth traits in female blue module and male turquoise module, in combination with the findings in WFM vs. WM and SFM vs. SM.
