# Supplementary figures and images for: Stat5b Regulates Sexually Dimorphic Gene Expression in Zebrafish Liver
Source: Front Physiol. 2018 May 31;9:676. doi: 10.3389/fphys.2018.00676 (PMC5990605; doi:10.3389/fphys.2018.00676)

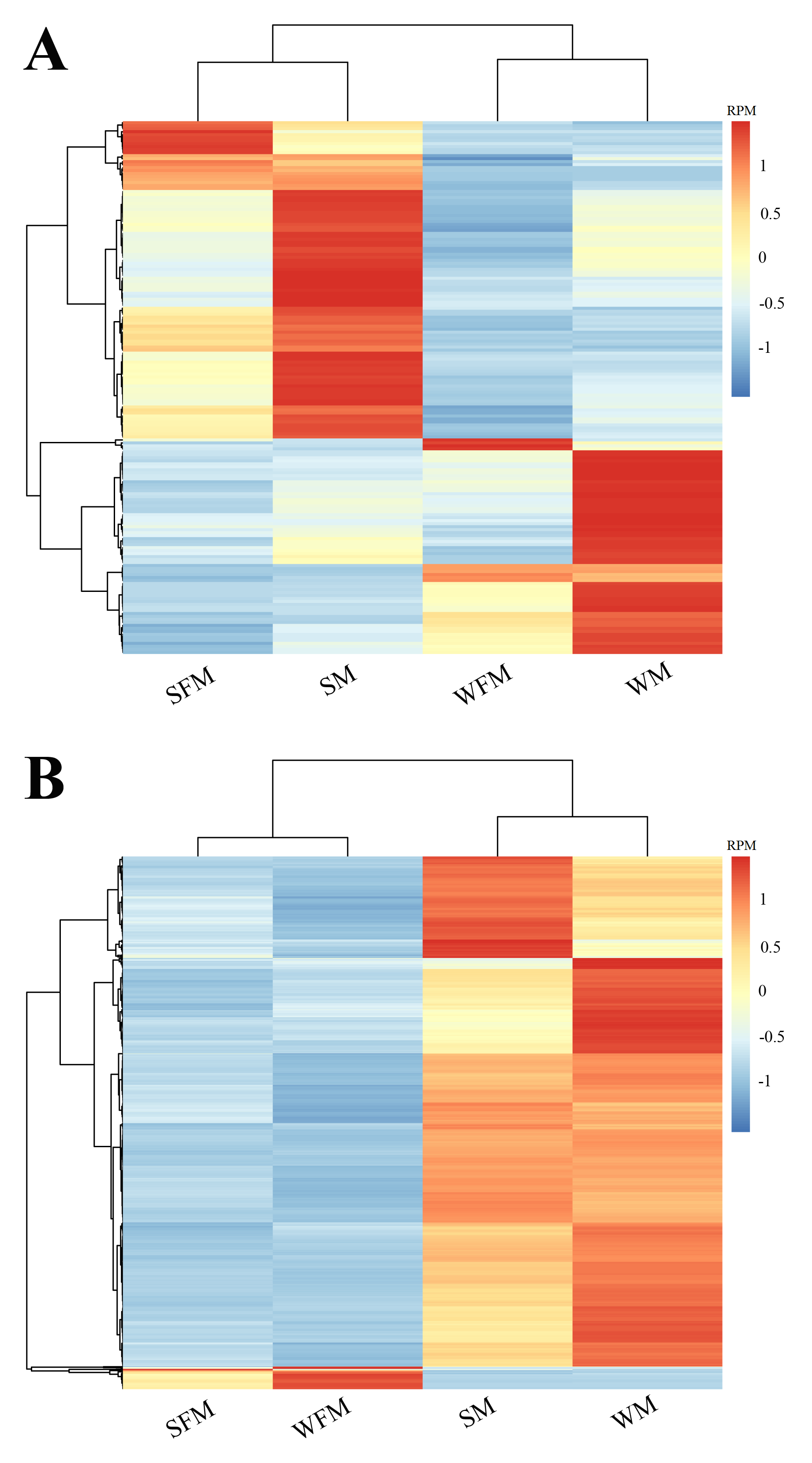

Supplement: FIGURE S1 — Differential gene expression analysis and clustering. Heat map of the DEGs among four comparisons (WFM vs. WM, SFM vs. SM, WFM vs. SFM, and WM vs. SM). (A) Common DEGs of WFM vs. SFM and WM vs. SM; (B). Common DEGs of WFM vs. WM and SFM vs. SM. Colors represent the summed RPM of three replicates after scaling and centering. [file Image_1.TIF]

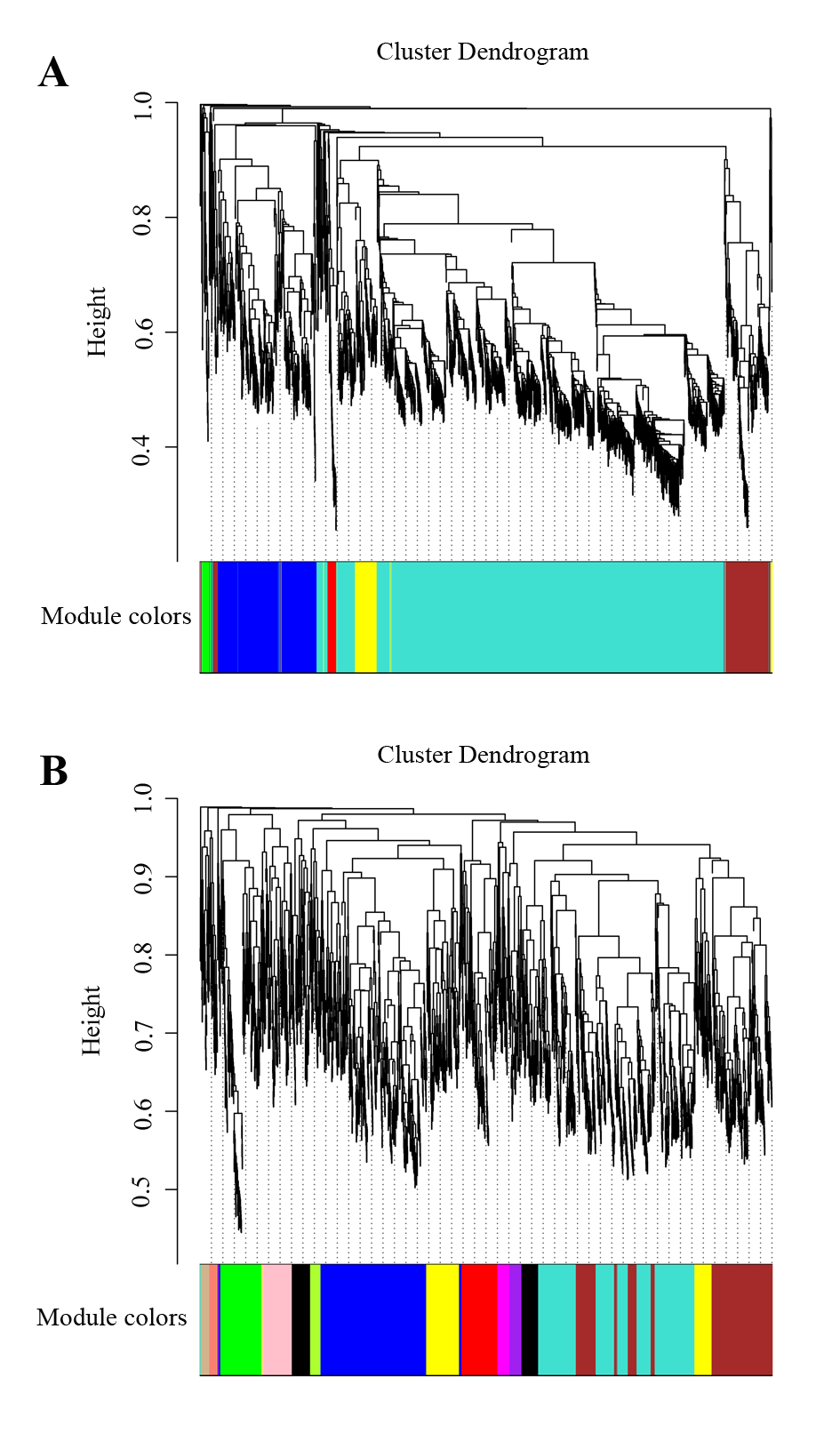

Supplement: FIGURE S2 — Cluster dendrogram with the WGCNA approach based on the correlation coefficients of DEGs between wildtype and stat5b-mutated livers in female (A) and male (B). The height shows the distance between two separate genes. Branches in the cluster dendrogram indicate the divided modules based on gene clustering result. Colored modules indicate the membership of separate module by combining modules with similar expression patterns. [file Image_2.TIF]

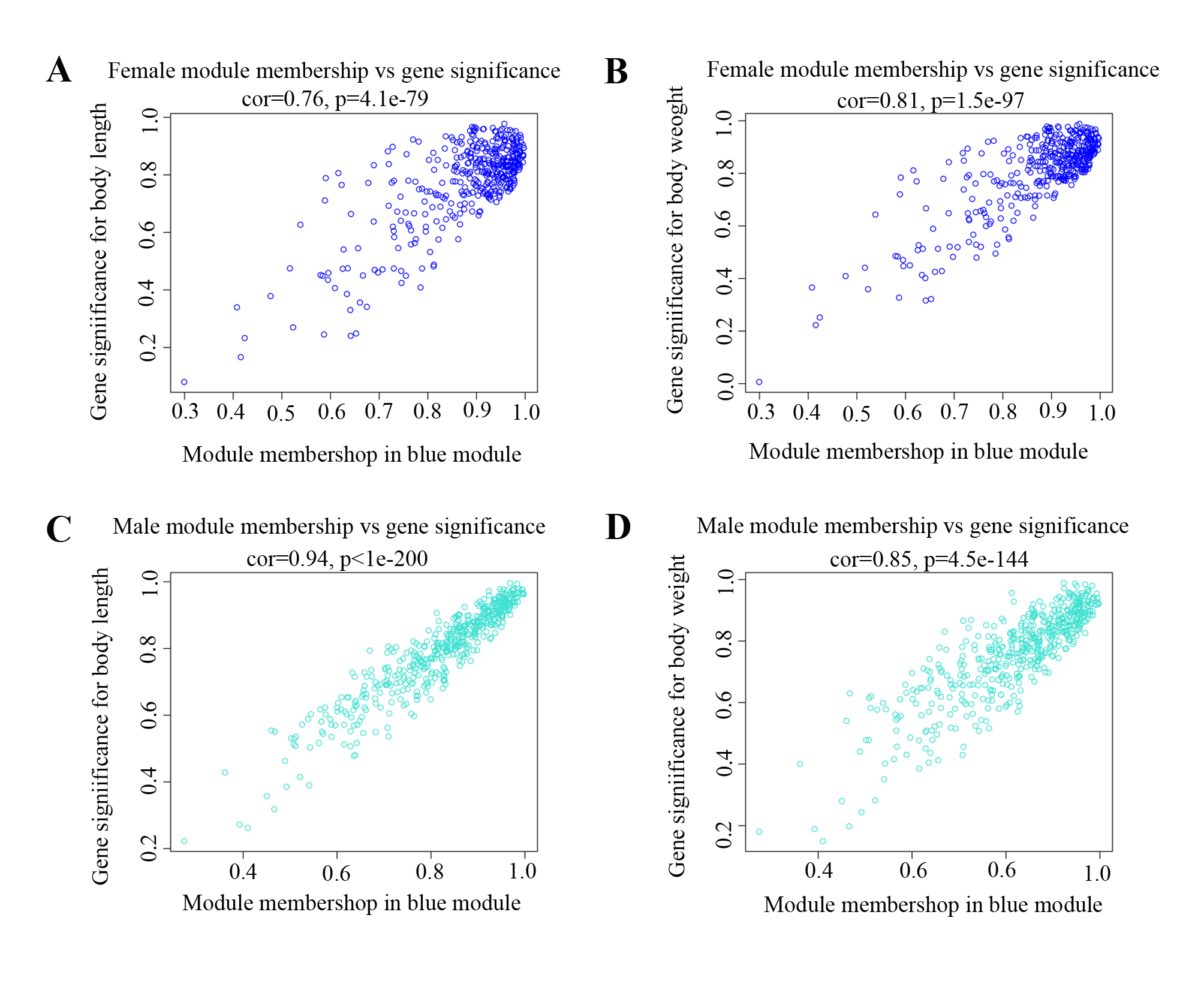

Supplement: FIGURE S3 — Module Membership vs. gene significance for growth trait. (A) Female module membership in blue module vs. gene significance for body length. (B) Female module membership in blue module vs. gene significance for body weight. (C) Male module membership in turquoise module vs. gene significance for body length. (D) Male module membership in turquoise module vs. gene significance for body weight. [file Image_3.TIF]
