# Supplementary material for: Stat5b Regulates Sexually Dimorphic Gene Expression in Zebrafish Liver
Source: Front Physiol. 2018 May 31;9:676. doi: 10.3389/fphys.2018.00676 (PMC5990605; doi:10.3389/fphys.2018.00676)
Supplement: TABLE S1 — The growth traits (body length and body weight) of 3-month-old zebrafish for transcriptome. [file Table_1.DOCX]

| Sample | Mean length (cm) | Mean lweight (g) |
| --- | --- | --- |
| SFM1 | 3.1 | 0.2601 |
| SFM2 | 3.05 | 0.2398 |
| SFM3 | 2.95 | 0.2295 |
| SM1 | 2.95 | 0.2218 |
| SM2 | 3.1 | 0.2524 |
| SM3 | 3 | 0.2321 |
| WFM1 | 3.35 | 0.3248 |
| WFM2 | 3.4 | 0.3451 |
| WFM3 | 3.3 | 0.3145 |
| WM1 | 3.3 | 0.2734 |
| WM2 | 3.4 | 0.294 |
| WM3 | 3.4 | 0.2837 |
