# Supplementary material for: Stat5b Regulates Sexually Dimorphic Gene Expression in Zebrafish Liver
Source: Front Physiol. 2018 May 31;9:676. doi: 10.3389/fphys.2018.00676 (PMC5990605; doi:10.3389/fphys.2018.00676)
Supplement: TABLE S2 — The information of primers used for qRT-PCR. [file Table_2.DOCX]

The primers used for qRT-PCR.

| Gene name | Forward primer | Reverse primer |
| --- | --- | --- |
| acp5a | AGTACAAGGCCACGGCTTAC | ACCACCCAATGTTGAGGCAT |
| acp5b | ATCGGTCATCACGGTCCAAC | ACGACGAACGAACTCCCATC |
| blnk | GATCAGCGCACTGAACTAGC | TTGAACAGGCTGTCGTCGTT |
| bmp2a | AACGCAGAGCAGGTTAGCAG | ACTGGAGCTGGACGGATCTT |
| bmp6 | GCCTTCCGGAACGAGACATT | CCACAACCTGCGAGATTCCA |
| ca2 | AGTGTCCTTCGTTGATGAT | GAGTCTGAATGTGCCTGTG |
| ctsk | GTGTGGACTGGCTCACTCTC | CTCCCAAATCGTCCGTCGAA |
| fosab | TTACCAGCCTTAACGCCGAC | TGGACCATCCACTGCAAGTC |
| ostm1 | CAAGGACTGCAAACCCCGAT | AGGGAACGAGCAACCAAAGT |
| vtg3 | CCTCAAACCAATACCTGTT | TTCTTTCATCTGTTACCTCC |
| moxd1 | GCGATGTCTTCGTCTGATA | GTGTGTCATTCTCTCTGCC |
| cyp1a | TATCGTAGTATCCGTGGCT | TTTACTGAAGCGTTCGTTG |
| cyp3c1 | TTTGTTGGCACGGCTTTAT | ACGGATTCGCCTCCACCTC |
| cyp20a1 | CTGAATGAAACCGTCCGCA | GACGACACCAAGGGCATAG |
| tgfb1a | AGTCGGGATTGATAAGTAAGC | AATGTCTGTGCGTTATTGTG |
| igfbp3 | AAGACGCTTCCGCATTCCA | TGTCTCCCTGTTTCCACCA |
| ghra | TGGCGATTACCTGGTGTCT | AGGAGGATTTGGAGGGAGG |
| ghrb | ACAACTCCCAGAGGAATGA | TTTTCTATGCTAAAGGACA |
| abca2 | GCTGCTTGTGGATGGACTA | TTTCATTGGTTTTCTCGGT |
| lepr | AATGGAGCCAACCTTTCTA | TGCCGTTCAGAAACCACAC |
| esr1 | AACCTTCCTCGCCACCTGC | GGTAGTATCCCACTGAAGC |
| vtg2 | TTCTCAAGGGCAACTACAA | CAGATACTAATGGAACGGT |
| col1a1a | GGTGCTTTTGGCAAGAGGA | CATTACGGTGCCGCTGTCG |
| greb1 | CTGGCCATCAGGATTCAATAAT | CCATTAGGGCCAGAGAGTAATG |
| igf2b | GTTGTATCATCGGTCTGGG | TTCATTCTTTGTGGCATCG |
| ef1a | GGCTGACTGTGCTGTGCTGATTG | CTTGTCGGTGGGACGGCTAGG |
