# Supplementary material for: Stat5b Regulates Sexually Dimorphic Gene Expression in Zebrafish Liver
Source: Front Physiol. 2018 May 31;9:676. doi: 10.3389/fphys.2018.00676 (PMC5990605; doi:10.3389/fphys.2018.00676)
Supplement: TABLE S3 — Summary of the sequencing data. The number 1, 2, and 3 represent three replicate samples of each group. [file Table_3.DOCX]

Raw data of the transcriptome

| Sample | length | Reads | Bases | Q20 (%) | Q30 (%) | GC (%) | N (ppm) |
| --- | --- | --- | --- | --- | --- | --- | --- |
| SFM1 | 150.00 | 68110826 | 10216623900 | 96.83 | 92.77 | 46.90 | 474.90 |
| SFM2 | 150.00 | 60385590 | 9057838500 | 96.24 | 91.65 | 46.89 | 477.34 |
| SFM3 | 150.00 | 56498872 | 8474830800 | 96.28 | 91.61 | 47.15 | 465.20 |
| SM1 | 150.00 | 60071298 | 9010694700 | 96.27 | 91.76 | 45.04 | 473.19 |
| SM2 | 150.00 | 71364092 | 10704613800 | 96.23 | 91.58 | 45.10 | 469.41 |
| SM3 | 150.00 | 60742790 | 9111418500 | 96.26 | 91.71 | 45.37 | 476.42 |
| WFM1 | 150.00 | 59807440 | 8971116000 | 96.43 | 91.72 | 46.53 | 355.62 |
| WFM2 | 150.00 | 52631312 | 7894696800 | 95.80 | 90.46 | 45.84 | 377.08 |
| WFM3 | 150.00 | 56942022 | 8541303300 | 95.78 | 90.41 | 45.23 | 378.54 |
| WM1 | 150.00 | 58365428 | 8754814200 | 95.82 | 90.48 | 45.08 | 376.49 |
| WM2 | 150.00 | 58213540 | 8732031000 | 95.38 | 89.47 | 45.42 | 283.43 |
| WM3 | 150.00 | 69331756 | 10399763400 | 96.01 | 90.87 | 44.88 | 377.04 |

Clean data of the transcriptome after filtering

| Sample | length | Reads | Bases | Q20 (%) | Q30 (%) | GC (%) | N (ppm) |
| --- | --- | --- | --- | --- | --- | --- | --- |
| SFM1 | 148.30 | 67204706 | 9966459331 | 97.44 | 93.52 | 46.95 | 84.12 |
| SFM2 | 147.94 | 59426980 | 8791519825 | 97.01 | 92.60 | 46.93 | 83.30 |
| SFM3 | 148.24 | 55656780 | 8250387968 | 96.95 | 92.45 | 47.20 | 83.63 |
| SM1 | 148.02 | 59127180 | 8751729933 | 97.01 | 92.69 | 45.07 | 83.46 |
| SM2 | 148.00 | 70322810 | 10407546483 | 96.95 | 92.47 | 45.11 | 83.30 |
| SM3 | 147.98 | 59828688 | 8853249283 | 96.99 | 92.62 | 45.41 | 84.35 |
| WFM1 | 148.01 | 59029722 | 8737073321 | 97.03 | 92.45 | 46.54 | 105.59 |
| WFM2 | 147.94 | 51678690 | 7645521822 | 96.57 | 91.39 | 45.85 | 80.71 |
| WFM3 | 148.00 | 55923966 | 8276812975 | 96.55 | 91.35 | 45.23 | 81.45 |
| WM1 | 148.01 | 57349860 | 8488488425 | 96.55 | 91.38 | 45.07 | 80.80 |
| WM2 | 147.72 | 57634606 | 8513905200 | 96.22 | 90.61 | 45.44 | 16.91 |
| WM3 | 147.87 | 68122664 | 10073104875 | 96.72 | 91.74 | 44.88 | 81.54 |
